# Left Ventricular Diastolic Dysfunction across Levels of Kidney Function: A Cross-Sectional Study Based on Routine Clinical Practice Data

**DOI:** 10.3390/jcm13175313

**Published:** 2024-09-08

**Authors:** Cindy P. Porras, Elisa Dal Canto, Anne-Mar L. van Ommen, M. Louis Handoko, Saskia Haitjema, Mark C. H. de Groot, Michiel L. Bots, Marianne C. Verhaar, Robin W. M. Vernooij

**Affiliations:** 1 Department of Nephrology and Hypertension, University Medical Center Utrecht, Room F03.204, Heidelberglaan 100, Postbus 85500, 3584CX Utrecht, The Netherlands; c.p.porrasacosta@umcutrecht.nl (C.P.P.); m.c.verhaar@umcutrecht.nl (M.C.V.); 2 Julius Center for Health Sciences and Primary Care, University Medical Center Utrecht, Utrecht University, Universiteitsweg 100, Postbus 85500, 3584CX Utrecht, The Netherlands; e.dalcanto@umcutrecht.nl (E.D.C.); m.l.bots@umcutrecht.nl (M.L.B.); 3 Laboratory of Experimental Cardiology, University Medical Center Utrecht, Utrecht University, Heidelberglaan 100, Postbus 85500, Postbus 85500, 3508GA Utrecht, The Netherlands; a.m.l.vanommen@umcutrecht.nl; 4 Department of Cardiology, Amsterdam University Medical Center, De Boelelaan 1118, Postbus 7057, 1007MB Amsterdam, The Netherlands; m.l.handoko@umcutrecht.nl; 5 Amsterdam Cardiovascular Sciences/Heart Failure and Arrhythmias, Amsterdam University Medical Center, Meibergdreef 9, 1105AZ Amsterdam, The Netherlands; 6 Department of Cardiology, University Medical Center Utrecht, Heidelberglaan 100, 3584CX Utrecht, The Netherlands; 7 Central Diagnostic Laboratory, Division Laboratories, Pharmacy and Biomedical Genetics, University Medical Center Utrecht, Utrecht University, Heidelberglaan 100, 3584CX Utrecht, The Netherlands; s.haitjema@umcutrecht.nl (S.H.); m.c.h.degroot-3@umcutrecht.nl (M.C.H.d.G.)

**Keywords:** diastolic dysfunction, kidney dysfunction, echocardiography

## Abstract

Left ventricular diastolic dysfunction (LVDD) commonly coexists with kidney dysfunction. In this study, we investigated the presence of abnormalities in echocardiography parameters indicative of LVDD across stages of kidney function. **Methods:** We selected patients who visited a university hospital and had a serum creatinine and echocardiography reported in their medical records. Participants were categorized based on their kidney function: normal (estimated glomerular filtration rate [eGFR] ≥ 90 mL/min/1.73 m^2^), mildly decreased (eGFR: 60–90), moderately decreased (eGFR: 30–60), and severely decreased (eGFR < 30). The relationship between kidney function and echocardiography parameters was examined using logistic and linear regressions. **Results:** Among 4022 patients (age: 66.5 years [SD: 12.1], 41% women), 26%, 50%, 20%, and 4% had a normal, mildly, moderately, and severely decreased kidney function, respectively. Compared to patients with normal kidney function, patients with mildly decreased kidney function had higher odds for an abnormal E/e′ ratio (OR: 1.51 [95% CI: 1.13, 2.02]). Patients with moderately decreased kidney function presented a higher risk of abnormal E/e′ (OR: 2.90 [95% CI: 2.08, 4.04]), LAVI (OR: 1.62 [95% CI: 1.13, 2.33]), TR velocity (OR: 2.31 [95% CI: 1.49, 3.57]), and LVMI (OR: 1.70 [95% CI: 1.31, 2.20]), while patients with severely decreased kidney function had higher odds for abnormal E/e′ (OR: 2.95 [95% CI: 1.68, 5.17]) and LVMI > 95 g/m^2^ in women or >115 g/m^2^ in men (OR: 2.07 [95% CI: 1.27, 3.38]). The linear regression showed a significant inverse association between eGFR and echocardiography parameters, meaning that with worse kidney function, the parameters for LVDD worsened as well. **Conclusions:** Abnormal echocardiography parameters of LVDD were present even in patients with mildly decreased kidney function. As the kidney function worsened, there was a gradual increase in the risk of abnormal parameters of LVDD.

## 1. Introduction 

Chronic kidney disease (CKD) is a significant public health concern, affecting over 10% of the global population [1]. CKD is associated with several systemic complications, among which cardiovascular disease (CVD) prevails [2], as approximately 50% of the patients with CKD have some form of CVD [3]. One of the most prominent cardiovascular complications present in patients with CKD is left ventricle diastolic dysfunction (LVDD), i.e., an impaired relaxation, filling, and/or distensibility of the left ventricle during diastole [4,5]. 

The coexistence of CKD and LVDD significantly increases the risk of heart failure, cardiovascular morbidity, and mortality, reflecting their impact on cardiovascular health [6,7,8]. LVDD is considered the preclinical stage of heart failure with preserved ejection fraction (HFpEF) and is commonly found in patients with CKD stages 4 and 5 [9,10,11]. Despite these important implications for patient care, existing research has primarily focused on patients with severe kidney dysfunction, often overlooking those with milder kidney dysfunction, which is more prevalent than severe kidney dysfunction. Furthermore, previous studies have typically examined a limited set of echocardiographic parameters (e.g., E/e′ ratio or E/A ratio) [8,12], consisted of relatively small sample sizes, or were not representative of clinical practice [13]. 

Traditionally, there were limited effective therapies specifically targeting LVDD and HFpEF, which might have diminished the perceived necessity of detailed diastolic function evaluation in clinical practice. In recent years, there have been significant developments in therapeutic options for HFpEF [14,15]. These advancements have shifted the landscape of treatment, making the assessment of diastolic function increasingly important. We hypothesize that abnormal echocardiography parameters that reflect diastolic function are present in patients with kidney dysfunction, already at mild stages of kidney dysfunction. Therefore, this cross-sectional study aimed to investigate echocardiographic parameters of LVDD in a real-world clinical setting among patients with various stages of kidney dysfunction. 

## 2. Materials and Methods 

The Strengthening the Reporting of Observational Studies in Epidemiology (STROBE) guidelines were used to ensure transparent reporting (Supplementary Material Table S1) [16].

### 2.1. Study Population

This cross-sectional study used electronic health records from the Utrecht Patient Oriented Database (UPOD), which comprises data of all patients treated at the University Medical Center Utrecht (UMC Utrecht) [17]. UPOD contains information on clinical characteristics, medication, and laboratory measurements collected as part of routine care since 2000 and has been described in detail elsewhere [18]. The study population consisted of adults from the outpatient departments of nephrology, cardiology, urology, cardiothoracic surgery, vascular medicine, vascular surgery, and neurology, treated at the UMC Utrecht between 2012 and 2022 with a documented estimated kidney function (i.e., serum creatinine) performed 31 days around the index date and a first echocardiography performed (both for any indication) up to 180 days after the index date. The index date was defined as the first visit to the outpatient department listed above. We excluded patients aged below 18 years, those who had received renal replacement therapies (i.e., peritoneal dialysis, hemodialysis, or kidney transplantation), and patients with a history of atrial fibrillation and mitral stenosis prior to the index date.

### 2.2. Determinants and Covariates

The patients’ estimated kidney function was based on the measurement of serum creatinine levels. We calculated the estimated glomerular filtration rate (eGFR) for all patients included in this study using the latest CKD-EPI 2021 creatinine formula without ethnicity [19]. 

Based on their eGFR, patients were categorized into four groups: normal kidney function (eGFR ≥ 90 mL/min/1.73 m^2^), mildly decreased kidney function (eGFR: 60–90), moderately decreased kidney function (eGFR: 30–60), and severely decreased kidney function (eGFR < 30). The normal kidney function group served as the reference group in all analyses.

We used data on history (i.e., blood pressure measurements), International Classification Disease (ICD) codes, diagnosis treatment combination (DBC) codes, medication (i.e., Anatomical Therapeutic Chemical Classification [ATC codes]), and laboratory measurements (e.g., glycated hemoglobin [HbA1c]) reported before the index date to define medical history. We defined hypertension as systolic blood pressure ≥ 140 mmHg and/or diastolic blood pressure ≥ 90 mmHg, a history of use of blood-pressure-lowering medication, or a history of hypertension as in medical records based on ICD–DBC codes. Diabetes mellitus (DM) was defined as HbA1c ≥ 48 mmol/mL, a history of use of glucose-lowering medication, or a history of DM in medical records, without distinction between DM types 1 and 2. The prevalence of CVD was based on a history of myocardial infarction, stroke, or transient ischemic attack, peripheral artery disease, renal artery stenosis, or abdominal aortic aneurysm using ICD–DBC codes reported in medical records. History of autoimmune disease, malignancy, and chronic obstructive pulmonary disease or asthma were defined using the ICD–DBC codes and medication registered in the electronic health records (for details see Supplementary Material Table S2). For all analyses, in patients without ICD–DBC codes, medication records, or laboratory measurements related to a history of comorbidities, we maintained a conservative approach and assumed that they did not have the comorbidities. 

### 2.3. Outcome of Interest 

Information on echocardiography parameters was extracted from the different echocardiographies reported in the electronic health records. We selected the parameters based on the guidelines for the assessment of LVDD from the American Society of Echocardiography (ASE) and the European Association of Cardiovascular Imaging (EACVI) [20,21]. We focused on the early mitral inflow velocity and mitral annular early diastolic velocity (E/e′ ratio), left atrial volume index (LAVI), tricuspid regurgitation (TR) velocity, relative wall thickness (RWT), left ventricle mass index (LVMI), and left ventricle posterior wall thickness (LVPWT). The cut-off values considered abnormal, based on the recommendation of the ASE and EACVI, were the following: E/e′ > 14, LAVI > 34 mL/m^2^, TR velocity > 2.8 m/s, RWT > 0.42, LVMI > 95 g/m^2^ in women and >115 g/m^2^ in men, and LVPWT > 1.2 cm. 

### 2.4. Statistical Analysis

The baseline characteristics were stratified and presented by estimated kidney function stage. Descriptive statistics were used to summarize the characteristics of the study population, and data were presented as counts and percentages (%) for categorical variables and a mean with standard deviation (SD) or median with interquartile range (IQR) for continuous variables, depending on the distribution. 

We assessed the relationship between estimated kidney function, as both continuous variables and categories (with normal kidney function as the reference group), and the presence of abnormal echocardiography parameters by using linear and logistic regression analyses, respectively. We estimated odds ratios (ORs), beta (β) estimates, and their 95% confidence intervals (95% CI). Three models were applied for all analyses, namely the crude model; Model 1, adjusted for age and sex; and Model 2, adjusted for age, sex, and history of DM, hypertension, CVD, and heart failure. To ensure accurate adjustment, we accounted for variables that may affect the relationship between eGFR and LVDD, as described by previous research on the topic (e.g., age, sex, hypertension, DM, CVD, and heart failure) [11,22,23]. We tested for interaction between kidney function and sex.

If missing values in the outcome of interest (echocardiography parameters) were present, we performed 25 imputations using the Multivariate Imputation by Chained Equations (MICE) method [21] and additionally repeated the analyses in complete cases (which only utilizes data from observations with complete information on the outcome) and available cases (which uses all available data on the outcome). 

### 2.5. Sensitivity Analyses

To assess the robustness of our findings, we conducted sensitivity analyses, repeating the analyses in patients with preserved (≥50%) and reduced (<50%) ejection fractions separately. We conducted the analyses using the three methods: complete cases, available cases, and imputed data. The results from the regression analyses presented in this manuscript are from imputed datasets. All statistical analyses were performed using R Studio (R version 4.3.0 [023-04-21 ucrt]) [24] and the MICE package [25]. 

## 3. Results 

### 3.1. Baseline Characteristics

We included 4022 patients (41.4% women). A total of 26% had normal kidney function, 50% had mildly decreased kidney function, 20% had moderately decreased kidney function, and 4% had severely decreased kidney function. Kidney function worsened as age increased in patients with normal kidney function, presenting a mean age of 61.8 (SD: 11.5), while those with severely decreased kidney function had a mean age of 71.0 years (SD: 12.6). Similarly, the prevalence of hypertension increased as kidney function worsened: 63% in patients with normal kidney function versus 85% in those with severely decreased kidney function. Patients with moderately decreased kidney function exhibited the highest prevalence of history of CVD and heart failure, 84% and 25%, respectively (see Table 1).

Regarding medication used within the cohort, metoprolol was prescribed to 40–46% of the population. When medications were classified by ATC codes, the proportion of patients using antithrombotic medication (ATC codes: B01) increased from 65% in patients with normal kidney function to 83% and 72% in patients with moderately and severely decreased kidney function, respectively. Similarly, agents acting on the renin–angiotensin system (ATC codes: C09) and diuretics (ATC codes: C03) were used by 50% and 36% and 70% and 79% of patients with normal and severely decreased kidney function, respectively. Supplementary Tables S3 and S4 detail the ten most frequently used medications and ATC codes within the cohort.

### 3.2. Echocardiography Parameters

**Figure 1:**
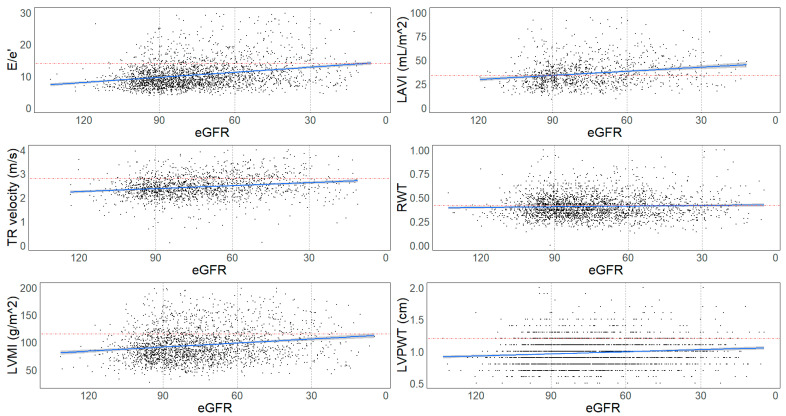
Echocardiography markers of LVDD at baseline scatter plot of eGFR against E/e′, left atrial volume index (LAVI), tricuspid regurgitation (TR) velocity, relative wall thickness (RWT), left ventricular mass index (LVMI), and left ventricular posterior wall thickness (LVPWT). eGFR: estimated glomerular filtration rate. Blue lines: regression line. Red lines: cut-off of normal value for each parameter. Vertical lines at 90, 60, and 30 identify the number of people in each kidney disease category: normal kidney function: eGFR ≥ 90 mL/min/1.73 m^2^; mildly decreased kidney function: eGFR ≥60–<90 mL/min/1.73 m^2^; moderately decreased kidney function: eGFR ≥30–<60 mL/min/1.73 m^2^; severely decreased kidney function: eGFR < 30 mL/min/1.73 m^2^.

The percentage of patients with abnormal echocardiography parameters was significantly higher as kidney function deteriorated. Almost 5% of the patients with normal kidney function had an E/e′ ratio (>14), and this percentage increased to 16.4% (*p*-value < 0.001) and 15.0% (*p*-value < 0.001) for those with moderate and severely decreased kidney function, respectively. Similar trends were observed for other echocardiography parameters. For instance, LAVI > 34 mL/m^2^ was present in 11.4% of participants with normal kidney function but increased to 16.3% (*p*-value < 0.001) for those with moderately decreased kidney function. The percentage of patients with TR velocity > 2.8 m/s increased from 4.4% in patients with normal kidney function to 7.6% (*p*-value = 0.001) in those with mildly decreased kidney function and 16.0% in those with moderately decreased kidney function. The proportion of individuals with RWT > 0.42 and LVMI (>95 g/m^2^ in women or >115 g/m^2^ in men) also progressively increased with declining kidney function. See Table 2 and Figure 1.

### 3.3. Association between Kidney Function and LVDD

We observed a significant inverse association between eGFR and E/e′ ratio (β: −0.85 [−1.1, −0.62), LAVI (β: −1.3 [−2.1, −0.56), TR velocity (β: −0.05 [−0.07, −0.03), and LVMI (β: −3.1 [−4.3, −1.8]) in the fully adjusted model. This indicates that as eGFR decreased by one standard deviation, LAVI, TR velocity, and LVMI increased and vice versa. We did not find an association between eGFR and RWT and LVPWT (see Table 3).

**Figure 2:**
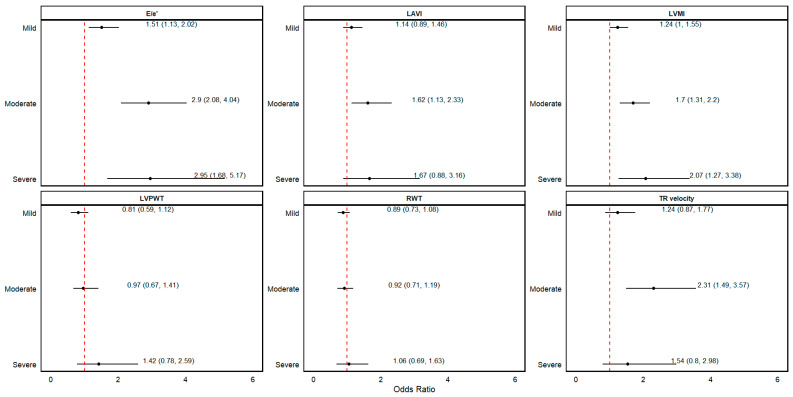
Reference group: normal kidney function: eGFR ≥ 90 mL/min/1.73 m^2^; mild kidney function: eGFR ≥60–<90 mL/min/1.73 m^2^; moderate kidney function: eGFR ≥30–<60 mL/min/1.73 m^2^; severe kidney function: eGFR < 30 mL/min/1.73 m^2^; TR velocity: tricuspid regurgitation; LAVI: left atrial volume index; RWT: relative wall thickness; LVMI: left ventricle mass index; LVPWT: left ventricle posterior wall thickness. The results presented are from Model 2: adjusted for age + sex + history of diabetes mellitus, hypertension, cardiovascular disease, and heart failure. Forest plots represent the odds of E/e′ > 14, LAVI > 34 mL/m^2^, TR velocity > 2.8 m/s, RWT > 0.42, LVMI > 95 g/m^2^ in women and >115 g/m^2^ in men, and LVPWT > 1.2 cm.

When eGFR was analyzed as clinical categories, we found that as eGFR decreased, distinct LVDD patterns became evident. Patients with a mildly decreased kidney function had higher odds of an abnormal E/e′ ratio (OR: 1.51 [1.13, 2.02]) compared to patients with normal kidney function. Patients with moderately decreased kidney function exhibited higher odds of an abnormal E/e′ ratio (OR: 2.90 [2.08, 4.04]), LAVI (OR: 1.62 [1.13, 2.33]), TR velocity (OR: 2.31 [1.49, 3.57]), and LVMI (OR: 1.70 [1.31, 2.20]). Patients with severely decreased kidney function presented higher odds of an abnormal E/e′ ratio (OR: 2.95 [1.68, 5.17]) and LVMI (OR: 2.07 [1.27, 3.38]) compared to patients with normal kidney function. (see Figure 2). Analyses including the interaction between kidney function and sex were not statistically significant; hence, we did not present analyses stratified by sex.

### 3.4. Sensitivity Analysis

Sensitivity analysis performed in patients with preserved ejection fraction Supplementary Material Tables S5–S8 revealed an inverse association of eGFR with E/e’, TR velocity, LVMI, and LVPWT, indicating that as kidney function increased, the E/e′ ratio, TR velocity, LVMI, and LVPWT decreased. On the other hand, when eGFR was analyzed as clinical categories, patients with moderately decreased kidney function showed a higher risk of an abnormal E/e′ ratio, TR velocity, and LVMI. Similarly, patients with severely decreased kidney function showed a higher risk of an abnormal E/e′ ratio and LVMI.

In patients with reduced ejection fraction, we found an inverse association of eGFR as a continuous variable with E/e′ ratio, LAVI, TR velocity, and LVMI. For categories of kidney function, patients with moderately decreased kidney function presented a higher risk of an abnormal E/e′, LAVI, and TR velocity, while those with severely decreased kidney function presented a higher risk of an abnormal E/e′ ratio compared to patients with normal kidney function.

## 4. Discussion

In this cross-sectional study of a large real-world data cohort of patients, we showed that as the kidney function worsened, there was a gradual increase in the presence of abnormal echocardiography parameters indicative of LVDD. Patients with mildly decreased kidney function were at a higher risk of having an abnormal E/e′ ratio, while those with moderately and severely decreased kidney function had even higher risks. Furthermore, patients with moderately and severely decreased kidney function displayed a higher risk of having abnormalities in other echocardiography markers, such as LAVI, TR velocity, and LVMI, compared to those with normal kidney function. In addition, we found consistent inverse relations between eGFR and the echocardiography parameters of LVDD, indicating that as eGFR decreased, the echocardiography parameters worsened and vice versa.

Left ventricular diastolic dysfunction and kidney dysfunction commonly coexist. This has been attributed to factors such as increased left ventricle stiffness, left ventricular hypertrophy (LVH), fibrosis, and shared risk factors [26]. Additionally, as kidney function deteriorates, diastolic function further deteriorates [10]. This may be due to several factors, such as volume overload that can cause increased filling pressures, leading to cardiac remodeling and hypertrophy [9] and other factors related to kidney dysfunction, such as neurohumoral alterations, inflammation, anemia, and mineral disorders, that are thought to lead to the development of LVDD [10,27]. LVDD is typically more common in older individuals and those with medical conditions such as hypertension and DM [23]. These risk factors were quite prevalent in our population. We found that abnormal echocardiography parameters were significantly associated with varying stages of kidney dysfunction, even after accounting for age, sex, and comorbidities. This suggests that kidney dysfunction itself has an independent relationship with cardiac abnormalities.

In 2017, Jain et al. described a significant relationship between worsening eGFR and the degree of LVDD assessed by E/e′ ratio, LVMI, and estimated right ventricular systolic pressure after adjustment for confounders in a retrospective cohort study in 2056 patients [28]. Additionally, our study showed that even in individuals with mildly decreased kidney function, there is a significantly higher risk of presenting abnormality in at least one echocardiography parameter. Furthermore, we aligned with previous research demonstrating a gradual development of abnormalities in echocardiography parameters of LVDD as kidney function deteriorates, with a more pronounced effect in patients with worse kidney function [11,28,29,30,31]. 

Kang et al. provided valuable insights into the bidirectional relationship between the heart and kidneys, demonstrating that a 1 unit increase in E/e′ was associated with a 2.1% increased hazard of renal event development, suggesting that patients with LVDD are at an increased risk of CKD progression [22]. Our study, however, highlights the reverse relationship, where reduced kidney function emerges as a significant risk factor for the development and progression of LVDD. These findings are not contradictory but rather illustrate the complex, bidirectional nature of the cardio-renal syndrome (CRS), where the heart and kidneys can both be initiators and targets of dysfunction.

We expanded on existing knowledge by assessing a wider range of echocardiographic parameters of LVDD, including TR velocity and RWT, which are useful to characterize cardiac function and structure, especially in the presence of elevated left ventricular filling pressures and hypertrophy [20,21]. Additionally, we used a larger sample size compared to other observational studies [11,32] and employed the new CKD-EPI 2021 formula without ethnicity, which is more accurate than the CKD-EPI 2009 and the modification of diet in renal disease (MDRD) equation, particularly for eGFR values greater than 60 mL/min/1.73 m^2^ [33].

### 4.1. Clinical Implications

The connection between LVDD and kidney dysfunction becomes more apparent as kidney disease progresses. This connection is linked to adverse cardiovascular outcomes and overall mortality, affecting around 50% of patients with an eGFR < 45 mL/min/1.73 m^2^ over five years [34,35]. Hence, assessment of diastolic function even in patients with mild kidney dysfunction is warranted. For years, the absence of specific therapies for LVDD and HFpEF might have reduced the perceived necessity for detailed diastolic function evaluation in clinical practice. The recent developments in therapeutic options for HFpEF, such as sodium–glucose cotransporters-2 (SGLT-2) inhibitors and glucagon-like peptide 1 (GLP-1) receptor agonists, have shifted the treatment landscape [14,15]. These agents have demonstrated substantial cardiovascular benefits, including reduction in body weight, heart failure hospitalizations, progression of kidney disease, and reduction in cardiovascular and all-cause mortality. These advancements make the evaluation of diastolic function in clinical practice increasingly important, as early identification of LVDD might serve as a first step to facilitate risk stratification for HFpEF and improve long-term prognosis. Yet, the decision to routinely assess diastolic function involves balancing clinical indications, symptoms, and the potential benefits against time and cost considerations. Future studies should explore the benefit of the early identification of LVDD in terms of impact on the prognosis.

### 4.2. Strengths and Limitations

The main strength of this study includes its large sample size of routine care data, which provided the opportunity to evaluate multiple echocardiography parameters in a large cohort of patients at different kidney function stages. However, some limitations need to be acknowledged. Although we assessed multiple parameters, as the ASE and the EACVI recommended, we focused on the abnormality of single echocardiography parameters. We acknowledge that a comprehensive multiparametric evaluation is ideal for a more accurate diagnostic of LVDD. However, the significant amount of missing data within the electronic health care records limited our ability to implement the algorithm proposed by Nagueh et al. [20] for the diagnostic of diastolic dysfunction. Consequently, while our findings provide valuable insights, they are limited by the inability to fully explore the broader multiparametric context of LVDD diagnosis. Second, there was a certain selection of patients who had at least a serum creatinine measurement and an echocardiography performed around the index date. Our cohort was derived from data collected in a university hospital, meaning that the findings may not be generalizable due to potential demographic differences. Third, due to the cross-sectional nature of the study, we cannot draw any conclusions about causality. Fourth, there may be potential unmeasured confounders that we have been unable to account for, such as body mass index (BMI) and smoking, which were not included in our analyses due to lack of data on BMI and smoking history in electronic care records. Finally, missing data posed a challenge, albeit mitigated through multiple imputation techniques, with no differences in the findings between complete case analysis and imputed data analysis.

## 5. Conclusions

Abnormal echocardiography parameters of LVDD are present in all patients with kidney dysfunction, even in those with mildly decreased kidney function. As kidney dysfunction worsened, more parameters were found to be abnormal, which aligns with the notion of the gradual development of LVDD in this population. Given new developments in therapeutic options for HFpEF, assessing diastolic function has become increasingly important. Therefore, clinicians should be aware that even mildly reduced kidney function is a risk factor for LVDD, and evaluation of diastolic function should be considered if patients are suspected of heart failure.

## Figures and Tables

**Table 1:** Baseline characteristics stratified by kidney function.

	Normal Kidney Function	Mildly Decreased Kidney Function	*p* *	Moderately Decreased Kidney Function	*p* **	Severely Decreased Kidney Function	*p* ***	*p* ****
N (%)	1050 (26)	2007 (50)		818 (20%)		147 (4%)		
Age (mean (SD)	61.83 (11.50)	66.95 (11.84)	<0.001	70.71 (11.43)	<0.001	71.01 (12.64)	<0.001	<0.001
Women (%)	437 (41.6)	850 (42.4)	0.726	310 (37.9)	0.114	69 (46.9)	0.257	0.08
Medical history								
Hypertension (%)	663 (63.1)	1271 (63.3)	0.951	568 (69.4)	0.005	125 (85.0)	<0.001	<0.001
Diabetes (%)	91 (8.7)	122 (6.1)	0.009	88 (10.8)	0.149	26 (17.7)	0.001	<0.001
CVD (%)	722 (68.8)	1525 (76.0)	<0.001	693 (84.7)	<0.001	117 (79.6)	0.010	<0.001
Heart failure (%)	130 (12.4)	316 (15.7)	0.014	208 (25.4)	<0.001	17 (11.6)	0.882	<0.001
COPD/asthma (%)	285 (27.1)	502 (25.0)	0.217	276 (33.7)	0.002	58 (39.5)	0.003	<0.001
Laboratories								
eGFR (ml/min/1.73 m^2^), median (IQR)	96.10 (92.92, 100.23)	77.30 (69.80, 84.13)	<0.001	49.01 (41.20, 55.18)	<0.001	22.36 (17.77, 27.27)	<0.001	<0.001
Potassium (mmol/L), mean (SD)	4.11 (0.32)	4.18 (0.34)	<0.001	4.32 (0.40)	<0.001	4.52 (0.43)	<0.001	<0.001
CRP (mg/L), median (IQR)	10.40 (2.30, 51.20)	8.30 (2.20, 48.50)	0.556	18.05 (3.80, 70.75)	<0.001	32.85 (6.78, 106.45)	<0.001	<0.001
Hba1c (mmol/mol), median (IQR)	39.00 (35.15, 47.25)	40.00 (36.00, 46.00)	0.197	42.00 (38.00, 50.38)	<0.001	48.50 (38.62, 61.75)	0.001	<0.001
Albumin (g/L), mean (SD)	36.84 (6.18)	37.23 (6.12)	0.346	36.01 (6.97)	0.102	33.65 (6.30)	<0.001	<0.001
Cholesterol (mmol/L), mean (SD)	4.99 (1.39)	4.83 (1.29)	0.013	4.55 (1.34)	<0.001	4.55 (1.34)	0.016	<0.001
HDL (mmol/L), median [IQR]	1.20 [1.00, 1.50]	1.20 [1.00, 1.55]	0.770	1.10 [0.90, 1.30]	<0.001	1.20 [0.95, 1.40]	0.057	<0.001
LDL (mmol/L), median [IQR]	2.80 [2.10, 3.60]	2.70 [2.00, 3.50]	0.209	2.40 [1.78, 3.10]	<0.001	2.20 [1.70, 3.00]	0.002	<0.001
Triglycerides (mmol/L), median [IQR]	1.60 [1.10, 2.30]	1.50 [1.10, 2.20]	0.556	1.70 [1.20, 2.60]	0.010	1.70 [1.20, 2.50]	0.001	0.005

Normal kidney function: eGFR ≥ 90 mL/min/1.73 m^2^; mildly decreased kidney function: eGFR ≥60–<90 mL/min/1.73 m^2^; moderately decreased kidney function: eGFR ≥30–<60 mL/min/1.73 m^2^; severely decreased kidney function: eGFR < 30 mL/min/1.73 m^2^; SD: standard deviation; IQR: interquartile range; CVD: cardiovascular disease; COPD: chronic obstructive pulmonary disease; eGFR: estimated glomerular filtration rate; HbA1c: glycated hemoglobin; CRP: C-reactive protein; HDL: high-density lipoprotein; LDL: low-density lipoprotein. * *p*-value reporting the comparison between normal and mildly decreased kidney function; ** *p*-value reporting the comparison between normal and moderately decreased kidney function; *** *p*-value reporting the comparison between normal and severely decreased kidney function; **** *p*-value reporting the comparison across all categories.

**Table 2:** Proportion of patients with abnormal echocardiography parameters.

	Normal Kidney Function	Mildly Decreased Kidney Function	*p* *	Moderately Decreased Kidney Function	*p* **	Severely Decreased Kidney Function	*p* ***	*p* ****
N	1050	2007		818		147		
E/e′ > 14 (%)	50 (4.8)	179 (8.9)	<0.001	134 (16.4)	<0.001	22 (15.0)	<0.001	<0.001
LAVI > 34 mL/m^2^	120 (11.4)	278 (13.9)	0.011	133 (16.3)	<0.001	16 (10.9)	0.001	<0.001
TR velocity > 2.8 m/s (%)	46 (4.4)	153 (7.6)	0.001	131 (16.0)	<0.001	12 (8.2)	0.128	<0.001
RWT > 0.42 (%)	256 (24.4)	495 (24.7)	0.985	190 (23.2)	0.003	41 (27.9)	0.205	0.004
LMVI >95 g/m^2^ in women or >115 g/m^2^ in men (%)	129 (12.3)	333 (16.6)	0.002	169 (20.7)	<0.001	32 (21.8)	<0.001	<0.001
LVPWT > 1.2 (%)	57 (5.4)	96 (4.8)	0.725	46 (5.6)	0.005	14 (9.5)	0.054	0.001

Normal kidney function: eGFR ≥ 90 mL/min/1.73 m^2^; mildly decreased kidney function: eGFR ≥60–<90 mL/min/1.73 m^2^; moderately decreased kidney function: eGFR ≥30–<60 mL/min/1.73 m^2^; severely decreased kidney function: eGFR < 30 mL/min/1.73 m^2^; TR velocity: tricuspid regurgitation; LAVI: left atrial volume index; RWT: relative wall thickness; LVMI: left ventricle mass index; LVPWT: left ventricle posterior wall thickness. * *p*-value reporting the comparison between normal and mildly decreased kidney function; ** *p*-value reporting the comparison between normal and moderately decreased kidney function; *** *p*-value reporting the comparison between normal and severely decreased kidney function; **** *p*-value reporting the comparison across all categories.

**Table 3:** Association between eGFR and LVDD markers.

eGFR	E/e′	LAVI (mL/m^2^)	TR velocity (m/s)	RWT	LMVI (g/m^2^)	LVPWT (cm)
	β (95% CI)	β (95% CI)	β (95% CI)	β (95% CI)	β (95% CI)	β (95% CI)
Crude						
**eGFR**	**−1.3 (−1.5, −1.1)**	**−2.7 (−3.4, −1.9)**	**−0.08 (−0.11, −0.06)**	−0.01 (−0.01, 0.00)	**−5.6 (−6.9, −4.3)**	**−0.02 (−0.03, −0.01)**
Model 1						
**eGFR**	**−1.1 (−1.3, −0.87)**	**−1.9 (−2.7, −1.1)**	**−0.06 (−0.09, −0.04)**	0.00 (0.00, 0.01)	**−4.8 (−6.1, −3.4)**	**−0.01 (−0.02, 0.00)**
Model 2						
**eGFR**	**−0.85 (−1.1, −0.62)**	**−1.3 (−2.1, −0.56)**	**−0.05 (−0.07, −0.03)**	0.00 (0.00, 0.01)	**−3.1 (−4.3, −1.8)**	−0.01 (−0.02, 0.00)

Statistically significant results are highlighted in bold. β: beta estimates per changes in one standard deviation in eGFR; CI: 95% confidence interval; eGFR mL/min/1.73 m^2^. TR velocity: tricuspid regurgitation; LAVI: left atrial volume index; RWT: relative wall thickness; LVMI: left ventricle mass index; LVPWT: left ventricle posterior wall thickness. Crude model; Model 1: adjusted for age and sex; Model 2: adjusted for Model 1 + history of diabetes mellitus, hypertension, cardiovascular disease, and heart failure.

## Data Availability

The data are based on information from confidential electronic health records. As such, data cannot be made publicly available. Requests for use of the data can be directed to the Utrecht Patient-Oriented Data-Based Project office (email: upod@umcutrecht.nl).

## Supplementary Materials

The following supporting information can be downloaded at https://www.mdpi.com/article/10.3390/jcm13175313/s1, Table S1: Strobe stable; Table S2: Disease definition; Table S3: Top 10 most common medication by kidney function; Table S4: Top 10 most common ATC categories by kidney function; Table S5: Sensitivity analysis logistic regression in patients with EF ≥ 50%; Table S6: Sensitivity analysis linear regression in patients with EF ≥ 50%; Table S7: Sensitivity analysis logistic regression in patients with EF < 50%; Table S8: Sensitivity analysis linear regression in patients with EF < 50%.
