# Supplementary material for: Left Ventricular Diastolic Dysfunction across Levels of Kidney Function: A Cross-Sectional Study Based on Routine Clinical Practice Data
Source: J Clin Med. 2024 Sep 8;13(17):5313. doi: 10.3390/jcm13175313 (PMC11396631; doi:10.3390/jcm13175313)
Supplement: Supplementary file 1 [file jcm-13-05313-s001.zip › jcm-3145925-supplementary.pdf]

Supplementary material

Table S1: Strobe stable

Table S2: Disease definition

Table S3: Top 10 most common medication by kidney function

Table S4: Top 10 most common ATC categories by kidney function

Table S5: Sensitivity analysis logistic regression in patients with EF ≥ 50%

Table S6: Sensitivity analysis linear regression in patients with EF ≥ 50%

Table S7: Sensitivity analysis logistic regression in patients with EF < 50%

Table S8: Sensitivity analysis linear regression in patients with EF <50%

Tables

Table S1: STROBE Statement—checklist of items that should be included in reports of observational studies

	Item No.	Recommendation	Page No.
**Title and abstract**	1	(*a*) Indicate the study's design with a commonly used term in the title or the abstract	2
		(*b*) Provide in the abstract an informative and balanced summary of what was done and what was found	3
**Introduction**			
Background/rationale	2	Explain the scientific background and rationale for the investigation being reported	7
Objectives	3	State specific objectives, including any prespecified hypotheses	7-8
**Methods**			
Study design	4	Present key elements of study design early in the paper	9
Setting	5	Describe the setting, locations, and relevant dates, including periods of recruitment, exposure, follow-up, and data collection	9
Participants	6	(*a*) *Cohort study*—Give the eligibility criteria, and the sources and methods of selection of participants. Describe methods of follow-up<br>*Case-control study*—Give the eligibility criteria, and the sources and methods of case ascertainment and control selection. Give the rationale for the choice of cases and controls<br>*Cross-sectional study*—Give the eligibility criteria, and the sources and methods of selection of participants	9
		(*b*) *Cohort study*—For matched studies, give matching criteria and number of exposed and unexposed<br>*Case-control study*—For matched studies, give matching criteria and the number of controls per case	
Variables	7	Clearly define all outcomes, exposures, predictors, potential confounders, and effect modifiers. Give diagnostic criteria, if applicable	9-10
Data sources/ measurement	8*	For each variable of interest, give sources of data and details of methods of assessment (measurement). Describe comparability of assessment methods if there is more than one group	9-10
Bias	9	Describe any efforts to address potential sources of bias	
Study size	10	Explain how the study size was arrived at	
Quantitative variables	11	Explain how quantitative variables were handled in the analyses. If applicable, describe which groupings were chosen and why	11
Statistical methods	12	(*a*) Describe all statistical methods, including those used to control for confounding	11
		(*b*) Describe any methods used to examine subgroups and interactions	12
		(*c*) Explain how missing data were addressed	11
		(*d*) *Cohort study*—If applicable, explain how loss to follow-up was addressed<br>*Case-control study*—If applicable, explain how matching of cases and controls was addressed<br>*Cross-sectional study*—If applicable, describe analytical methods taking account of sampling strategy	
		(*e*) Describe any sensitivity analyses	12

**Results**			
Participants	13*	(a) Report numbers of individuals at each stage of study—eg numbers potentially eligible, examined for eligibility, confirmed eligible, included in the study, completing follow-up, and analysed	13
		(b) Give reasons for non-participation at each stage	
		(c) Consider use of a flow diagram	

Descriptive data	14*	(a) Give characteristics of study participants (e.g. demographic, clinical, social) and information on exposures and potential confounders	13
		(b) Indicate number of participants with missing data for each variable of interest	
		(c) *Cohort study*—Summarise follow-up time (e.g. average and total amount)	
Outcome data	15*	*Cohort study*—Report numbers of outcome events or summary measures over time	
		Case-control study—Report numbers in each exposure category, or summary measures of exposure	
		Cross-sectional study—Report numbers of outcome events or summary measures	13-14
Main results	16	(*a*) Give unadjusted estimates and, if applicable, confounder-adjusted estimates and their precision (e.g. 95% confidence interval). Make clear which confounders were adjusted for and why they were included	22
		(*b*) Report category boundaries when continuous variables were categorized	13-21-23
		(*c*) If relevant, consider translating estimates of relative risk into absolute risk for a meaningful time period	
Other analyses	17	Report other analyses done—e.g. analyses of subgroups and interactions, and sensitivity analyses	14-supp material
**Discussion**			
Key results	18	Summarise key results with reference to study objectives	15
Limitations	19	Discuss limitations of the study, taking into account sources of potential bias or imprecision. Discuss both direction and magnitude of any potential bias	17
Interpretation	20	Give a cautious overall interpretation of results considering objectives, limitations, multiplicity of analyses, results from similar studies, and other relevant evidence	16
Generalisability	21	Discuss the generalisability (external validity) of the study results	17
**Other information**			
Funding	22	Give the source of funding and the role of the funders for the present study and, if applicable, for the original study on which the present article is based	18

Table S2: Disease definition

**Disease**	Definition
Diabetes type 1	1. Age of diabetes onset <40 years AND<br>2. Use of insulin (within 1 year of diagnosis?)<br>a. ATC A10A (Diabetes drugs)
Diabetes type 2	1. HbA1C ≥48 mmol/mol OR<br>2. Use of glucose-lowering medication OR<br>a. ATC_A10<br>3. History of diabetes in medical record (ICD-DBC codes E10-E14)
Hypertension	1. Blood pressure >140/90 mmHG OR<br>2. Use of blood-pressure lowering medication: ATC codes OR<br>a. ATC_C02 (antihypertensives)<br>b. ATC_C03 (diuretics)<br>c. ATC_C07 (beta-blockers)<br>d. ATC_C08 (calcium channel blockers)<br>e. ATC_C09 (RAAS-inhibitors)<br>3. History of hypertension in medical record (ICD-DBC codes I10-I13, I15)
Heart failure	1. History of heart failure in medical records ICD-DBC codes (I09.9, I25.5, I42.0, I42.5–I42.9, I43, I50)
Atherosclerotic cardiovascular disease	1. Medical history ICD-DBC codes:<br>a. Myocardial infarction: I21-I22<br>b. Stroke/TIA: G45.0-G45.3, G45.8-G45.9, H34.1, I60, I61, I63, I64<br>c. Angina + medicatation ATC_B01AC<br>d. PAD: I70.2, I73.9<br>e. Carotid artery disease: I65.2<br>f. Renal artery stenosis: I70.1, Q27.1<br>g. Vertebral artery stenosis<br>h. AAA: I71.2<br>2. Use of antiplatelets<br>a. ATC_B01AC
Lung disease	1. COPD/asthma/other lung disease ICD-DBC codes:<br>a. Asthma: J45<br>b. COPD: I27.8, I27.9, J40–J44, J46-J47, J60–J67, J68.4, J70.1, J70.3<br>2. Use of SAMA/SABA/ICS/LABA/LAMA<br>a. ATC_R03 (drugs for asthma/COPD)

Table S3: Top 10 most common medication by kidney function

Medication	Normal kidney function (n= 1050)	Mildly decreased kidney function (n= 2007)	Moderately decreased kidney function (n= 818)	Severely decreased kidney function (n= 147)
Metoprolol (%)	40.7	42.6	45.8	45.9
Dalteparine (%)	28.3	24.8	28.0	37.8
Carbasalate calcium (%)	25.2	26.8	25.5	19.6
Furosemide (%)	24.2	29.8	49.0	67.6
Acetylsalicylic acid (%)	22.3	25.5	25.7	23.6
Perindopril (%)	18.6	20.2	24.5	22.6
Acenocoumarol (%)	18.4	22.8	34.5	27.7
Amlodipine (%)	16.3	16.5	19.6	33.8
Clopidogrel (%)	13.3	17.3	-	-
Metformine (%)	11.8	-	17.5	-
Spironolactone (%)	-	15.4	29.0	20.9
Insulin aspart (%)	-	-	-	25.7

Table S4: Top 10 most common ATC categories by kidney function

ATC codes	Normal kidney function (n= 1050)	Mildly decreased kidney function (n= 2007)	Moderately decreased kidney function (n= 818)	Severely decreased kidney function (n= 147)
B01: Antithrombotic agents (%)	65.4	72.8	82.9	74.3
C09: Agents acting on the renin-angiotensin system (%)	50.4	57.1	72.0	69.6
C03: Diuretics (%)	35.7	45.5	69.4	79.1
C07: Beta blocking agents (%)	50.6	56.0	62.5	59.5
C08: Calcium channel blockers (%)	24.3	25.0	28.4	39.9
R03: Drugs for obstructive airway diseases (%)	24.8	23.0	31.2	38.5
H02: Corticoids for systemic use (%)	23.3	20.3	20.8	36.5
A10: Drugs used in diabetes (%)	17.8	17.5	26.6	37.8
L04: Immunosuppressants (%)	9.1	6.4	5.5	12.2
L01: Antineoplastic agents (%)	5.7	3.9	3.1	6.1

Table S5: Association between kidney function stages and LVDD markers patients with EF ≥ 50%

	**E/e'** (> 14)	**LAVI** (>34ml/m^2)	**TR velocity** (>2.8m/s)	**RWT** (>0.42)	**LMVI** (>95g/m^2 or >115g/m^2)*	**LVPWT** (>1.2cm)
	OR (95% CI)	OR (95% CI)	OR (95% CI)	OR (95% CI)	OR (95% CI)	OR (95% CI)
**Crude**						
Normal	ref	ref	ref	ref	ref	ref
Mildly decreased	**1.79 (1.19, 2.69)**	**1.34 (1.04, 1.73)**	1.41 (0.93, 2.12)	1.10 (0.90, 1.36)	**1.37 (1.04, 1.80)**	0.94 (0.66, 1.34)
Moderately decreased	**3.78 (2.46, 5.83)**	**1.96 (1.35, 2.84)**	**3.12 (1.77, 5.50)**	**1.59 (1.19, 2.12)**	**2.08 (1.44, 3.00)**	1.49 (0.97, 2.28)
Severely decreased	**4.48 (2.13, 9.44)**	1.62 (0.80, 3.28)	1.60 (0.59, 4.32)	**1.93 (1.15, 3.25)**	**2.37 (1.33, 4.21)**	**2.42 (1.18, 4.97)**
**Model 1**						
Normal	ref	ref	ref	ref	ref	ref
Mildly decreased	1.34 (0.88, 2.05)	1.09 (0.83, 1.44)	1.14 (0.75, 1.71)	0.92 (0.74, 1.14)	1.16 (0.87, 1.54)	0.76 (0.52, 1.11)
Moderately decreased	**2.42 (1.54, 3.81)**	1.35 (0.88, 2.07)	**2.20 (1.23, 3.93)**	1.15 (0.85, 1.57)	**1.61 (1.10, 2.35)**	1.02 (0.65, 1.61)
Severely decreased	**2.98 (1.34, 6.64)**	1.19 (0.57, 2.51)	1.15 (0.43, 3.10)	1.50 (0.88, 2.55)	**1.87 (1.02, 3.40)**	1.80 (0.85, 3.80)
**Model 2**						
Normal	ref	ref	ref	ref	ref	ref
Mildly decreased	1.32 (0.87, 2.03)	1.07 (0.81, 1.41)	1.13 (0.74, 1.70)	0.93 (0.75, 1.15)	1.15 (0.86, 1.53)	0.76 (0.51, 1.12)
Moderately decreased	**2.22 (1.39, 3.52)**	1.29 (0.83, 2.00)	**2.12 (1.18, 3.78)**	1.14 (0.83, 1.55)	**1.49 (1.01, 2.20)**	0.95 (0.60, 1.49)
Severely decreased	**2.73 (1.20, 6.23)**	1.23 (0.58, 2.61)	1.09 (0.39, 3.00)	1.36 (0.79, 2.32)	1.81 (0.98, 3.37)	1.61 (0.76, 3.43)

Normal kidney function: eGFR ≥90 mL/min/1.73m^2
Mildly decreased kidney function: eGFR ≥60 - <90 mL/min/1.73m^2
Moderately decreased kidney function: eGFR ≥30 - <60 mL/min/1.73m^2
Severely decreased kidney function: eGFR<30 mL/min/1.73m^2
OR: odd ratio; CI: 95% confidence interval: TR velocity: tricuspid regurgitation; LAVI: left atrial volume index; RWT: relative wall thickness;
*LVMI: left ventricle mass index > 95g/m^2 in females or >115g/m^2 in males ; LVPWT: left ventricle posterior wall thickness.
F: female; M: male
Crude model
Model 1: adjusted for age and sex.
Model 2: adjusted for Model 1 + history of diabetes mellitus, hypertension, cardiovascular disease, and heart failure.

Table S6: Association between eGFR and LVDD markers patients with EF >50%

**eGFR**	**E/e'**	**LAVI (ml/m2)**	**TR velocity (m/s)**	**RWT**	**LMVI (g/m^2)**	**LVPWT (cm)**
	β (95% CI)	β (95% CI)	β (95% CI)	β (95% CI)	β (95% CI)	β (95% CI)
Crude						
**eGFR**	**-0.94 (-1.1, -0.74)**	**-1.5 (-2.3, -0.63)**	**-0.06 (-0.09, -0.03)**	**-0.02 (-0.02, -0.01)**	**-2.9 (-4.2, -1.7)**	**-0.03 (-0.04, -0.02)**
Model 1						
**eGFR**	**-0.63 (-0.83, -0.43)**	-0.51 (-1.4, 0.41)	**-0.03 (-0.06, 0.00)**	-0.01 (-0.01, 0.00)	**-1.7 (-3.0, -0.35)**	**-0.01 (-0.03, 0.00)**
Model 2						
**eGFR**	**-0.56 (-0.76, -0.35)**	-0.44 (-1.3, 0.45)	**-0.03 (-0.06, 0.00)**	-0.01 (-0.01, 0.00)	**-1.3 (-2.6, -0.07)**	**-0.01 (-0.02, 0.00)**

β: Beta estimates per changes in one-standard deviation in eGFR; CI: 95% confidence interval; eGFR mL/min/1.73m2. TR velocity: tricuspid regurgitation; LAVI: left atrial volume index; RWT: relative wall thickness; LVMI: left ventricle mass index; LVPWT: left ventricle posterior wall thickness.
Crude model
Model 1: adjusted for age and sex.
Model 2: adjusted for Model 1 + history of diabetes mellitus, hypertension, cardiovascular disease, and heart failure.

Table S7: Association between kidney function stages and LVDD markers patients with EF <50%

	**E/e'** (> 14)	**LAVI** (>34ml/m^2)	**TR velocity** (>2.8m/s)	**RWT** (>0.42)	**LMVI** (>95g/m^2 or >115g/m^2)*	**LVPWT** (>1.2cm)
	OR (95% CI)	OR (95% CI)	OR (95% CI)	OR (95% CI)	OR (95% CI)	OR (95% CI)
**Crude**						
Normal	ref	ref	ref	ref	ref	ref
Mildly decreased	**1.70 (1.11, 2.59)**	1.41 (0.94, 2.11)	1.50 (0.89, 2.52)	0.97 (0.63, 1.47)	1.34 (0.94, 1.90)	1.02 (0.53, 1.94)
Moderately decreased	**3.75 (2.42, 5.81)**	**2.64 (1.56, 4.47)**	**2.90 (1.71, 4.90)**	0.91 (0.58, 1.45)	**1.88 (1.25, 2.84)**	1.22 (0.60, 2.50)
Severely decreased	**3.08 (1.42, 6.66)**	2.56 (0.94, 6.92)	2.23 (0.98, 5.09)	1.46 (0.72, 2.95)	1.95 (0.87, 4.37)	1.66 (0.54, 5.06)
**Model 1**						
Normal	ref	ref	ref	ref	ref	ref
Mildly decreased	**1.61 (1.05, 2.47)**	1.25 (0.83, 1.89)	1.37 (0.82, 2.31)	0.82 (0.53, 1.28)	1.31 (0.92, 1.87)	0.94 (0.49, 1.82)
Moderately decreased	**3.44 (2.19, 5.39)**	**2.17 (1.27, 3.70)**	**2.52 (1.48, 4.30)**	0.70 (0.44, 1.12)	**1.81 (1.18, 2.79)**	1.08 (0.53, 2.23)
Severely decreased	**2.65 (1.21, 5.82)**	1.99 (0.73, 5.41)	1.87 (0.81, 4.33)	0.97 (0.47, 2.02)	1.75 (0.76, 4.03)	1.44 (0.47, 4.43)
**Model 2**						
Normal	ref	ref	ref	ref	ref	ref
Mildly decreased	**1.57 (1.02, 2.44)**	1.18 (0.77, 1.82)	1.32 (0.78, 2.23)	0.86 (0.55, 1.36)	1.23 (0.85, 1.78)	0.95 (0.49, 1.86)
Moderately decreased	**3.08 (1.93, 4.93)**	**1.90 (1.10, 3.29)**	**2.25 (1.29, 3.91)**	0.80 (0.49, 1.30)	1.54 (0.98, 2.41)	1.11 (0.53, 2.32)
Severely decreased	**2.52 (1.10, 5.80)**	2.16 (0.76, 6.15)	1.82 (0.76, 4.36)	0.93 (0.44, 1.95)	1.81 (0.77, 4.27)	1.32 (0.43, 4.11)

Normal kidney function: eGFR ≥90 mL/min/1.73m^2
Mildly decreased kidney function: eGFR ≥60 - <90 mL/min/1.73m^2
Moderately decreased kidney function: eGFR ≥30 - <60 mL/min/1.73m^2
Severely decreased kidney function: eGFR<30 mL/min/1.73m^2
OR: odd ratio; CI: 95% confidence interval: TR velocity: tricuspid regurgitation; LAVI: left atrial volume index; RWT: relative wall thickness;
*LVMI: left ventricle mass index > 95g/m^2 in females or >115g/m^2 in males ; LVPWT: left ventricle posterior wall thickness.
F: female; M: male
Crude model
Model 1: adjusted for age and sex.
Model 2: adjusted for Model 1 + history of diabetes mellitus, hypertension, cardiovascular disease, and heart failure.

Table S8: Association between eGFR and LVDD markers patients with EF <50%

eGFR	E/e'	LAVI (ml/m2)	TR velocity (m/s)	RWT	LMVI (g/m²)	LVPWT (cm)
	β (95% CI)	β (95% CI)	β (95% CI)	β (95% CI)	β (95% CI)	β (95% CI)
Crude						
**eGFR**	**-1.4 (-1.8, -0.92)**	**-2.7 (-4.0, -1.5)**	**-0.09 (-0.13, -0.06)**	0.00 (-0.01, 0.01)	**-4.8 (-7.7, -1.9)**	-0.01 (-0.03, 0.00)
Model 1						
**eGFR**	**-1.3 (-1.7, -0.77)**	**-2.1 (-3.4, -0.87)**	**-0.08 (-0.11, -0.04)**	0.01 (0.00, 0.01)	**-4.7 (-7.7, -1.6)**	-0.01 (-0.02, 0.01)
Model 2						
**eGFR**	**-1.1 (-1.5, -0.59)**	**-1.8 (-2.9, -0.57)**	**-0.07 (-0.11, -0.04)**	0.00 (0.00, 0.01)	**-3.5 (-6.4, -0.57)**	-0.01 (-0.02, 0.01)

β: Beta estimates per changes in one-standard deviation in eGFR; CI: 95% confidence interval; eGFR mL/min/1.73m2. TR velocity: tricuspid regurgitation; LAVI: left atrial volume index; RWT: relative wall thickness; LVMI: left ventricle mass index; LVPWT: left ventricle posterior wall thickness.
Crude model
Model 1: adjusted for age and sex.
Model 2: adjusted for Model 1 + history of diabetes mellitus, hypertension, cardiovascular disease, and heart failur
